# Defective Resection at DNA Double-Strand Breaks Leads to *De Novo* Telomere Formation and Enhances Gene Targeting

**DOI:** 10.1371/journal.pgen.1000948

**Published:** 2010-05-13

**Authors:** Woo-Hyun Chung, Zhu Zhu, Alma Papusha, Anna Malkova, Grzegorz Ira

**Affiliations:** 1 Department of Molecular and Human Genetics, Baylor College of Medicine, Houston, Texas, United States of America; 2 Department of Biology, School of Science, Indiana University–Purdue University Indianapolis, Indianapolis, Indiana, United States of America; National Cancer Institute, United States of America

## Abstract

The formation of single-stranded DNA (ssDNA) at double-strand break (DSB) ends is essential in repair by homologous recombination and is mediated by DNA helicases and nucleases. Here we estimated the length of ssDNA generated during DSB repair and analyzed the consequences of elimination of processive resection pathways mediated by Sgs1 helicase and Exo1 nuclease on DSB repair fidelity. In wild-type cells during allelic gene conversion, an average of 2–4 kb of ssDNA accumulates at each side of the break. Longer ssDNA is formed during ectopic recombination or break-induced replication (BIR), reflecting much slower repair kinetics. This relatively extensive resection may help determine sequences involved in homology search and prevent recombination within short DNA repeats next to the break. In *sgs1*Δ *exo1*Δ mutants that form only very short ssDNA, allelic gene conversion decreases 5-fold and DSBs are repaired by BIR or *de novo* telomere formation resulting in loss of heterozygosity. The absence of the telomerase inhibitor, PIF1, increases *de novo* telomere pathway usage to about 50%. Accumulation of Cdc13, a protein recruiting telomerase, at the break site increases in *sgs1*Δ *exo1*Δ, and the requirement of the Ku complex for new telomere formation is partially bypassed. In contrast to this decreased and alternative DSB repair, the efficiency and accuracy of gene targeting increases dramatically in *sgs1*Δ *exo1*Δ cells, suggesting that transformed DNA is very stable in these mutants. Altogether these data establish a new role for processive resection in the fidelity of DSB repair.

## Author Summary

Chromosomal breaks occur spontaneously or are induced by ionizing radiation and many chemotherapeutic drugs. DNA double-strand breaks are processed by nucleases and helicases in yeast and human to generate single-stranded DNA that is then used for repair by recombination with homologous chromosome. Single-stranded DNA at chromosomal breaks also constitutes a signal for cells to arrest cell cycle progression until the DNA damage is repaired. This study examines the consequences of elimination of enzymes that process chromosomal breaks to single-stranded DNA on the fidelity of repair and genome stability in the model organism yeast. Mutants deficient in these enzymes often fail to repair the breaks by homologous recombination and instead add new telomeres at the breaks. Formation of new telomeres is associated with partial loss of the chromosome arm distal from the break. Such chromosomal aberrations were frequently observed in tumor cells and are responsible for about 10% of human genomic disorders resulting from chromosomal abnormalities. We also observed that elimination of enzymes that process chromosomal breaks into single-stranded DNA greatly stimulates genome manipulation by gene targeting, suggesting that transformed DNA is also a substrate for degradation by these enzymes. We discuss the possibility of using a similar approach in mammalian cells where gene targeting is inaccurate and less efficient when compared to yeast.

## Introduction

Homologous recombination is a major mechanism of repair of both DNA double-strand breaks (DSBs) and gaps that occur spontaneously or are induced by endonucleases, radiation or radiomimetic agents. Most of the enzymes involved in recombination are conserved among bacteria, yeast and human [reviewed in [1], [2]]. A key protein in recombination called Rad51 mediates DNA strand exchange between a damaged DNA molecule and a homologous intact DNA template. Rad51 forms a nucleoprotein filament on 3′ single-stranded DNA (ssDNA) that is capable of a genome-wide search for a homologous template sequence and subsequent strand invasion. Upon strand invasion, 3′ ends initiate new DNA synthesis that allows recovery of lost information at the site of the DSB. Subsequent resolution of the recombining molecules forms a final product that in mitotic cells typically is a noncrossover.

A necessary prerequisite of Rad51 filaments is the formation of 3′ ssDNA tails at DNA breaks. In budding yeast, the Mre11/Rad50/Xrs2 (MRX) complex together with Sae2 is responsible for the initiation of resection while two nucleases, Exo1 and Dna2 together with the Sgs1/Top3/Rmi1 (STR) complex form long ssDNA at DSBs [3], [4], [5]. Similar pathways of resection operate at yeast telomeres [6]. A two step mechanism in which mre11 and rad50 homologues play an initial role has also been described in the archeal organism *Pyrococcus furiosus* [7]. Human orthologs of these proteins, Mre11-Rad50-Nbs1 (MRN) with CtIP, Exo1 and BLM, play similar roles in 5′ strand resection [3], [8], [9]. In *Xenopus laevis,* Dna2 also processes DSB ends [10]. Human Dna2 has an important role in the maintenance of mitochondrial DNA [11], yet its nuclear role remains to be determined [12].

Resection of the 5′ strand is highly regulated by DNA damage checkpoint proteins, chromatin remodeling factors and cyclin-dependent kinase. DNA damage checkpoint proteins exhibit complex interactions with enzymes involved in resection because they both stimulate and later limit resection [[13] and reviewed in [14], [15]]. Cell cycle control of resection influences the choice between DSB repair pathways: nonhomologous end joining (NHEJ) or homologous recombination (HR) [16], [17], [18]. The extent of resection at DSBs is clearly regulated. However, how resection proceeds during DSB repair and the consequences of excess or limited resection on the fidelity of repair are not known.

Most estimations of end resection in yeast were made using DSBs generated by either the HO or I-SceI endonucleases that cannot be repaired by gene conversion because homologous sequences are deleted [e.g. [19], [20]]. Resection of such breaks is unlimited and proceeds at about 4 kb per hour. An alternative approach used to study resection utilized single strand annealing (SSA), a repair process relying on extensive resection and annealing between distant direct DNA repeats [e.g. [21]]. While these assays were very useful in identifying proteins involved in resection it remains unknown how resection proceeds when the break is being repaired by the most natural pathway, gene conversion. It is important to examine the resection at DSBs that are repaired normally because resection determines sequences that are used for homology search and repair. Here for the first time, we estimated the length of ssDNA generated during DSB repair using several assays with different kinetics of repair - allelic and ectopic gene conversion and break-induced replication (BIR). We demonstrate that about 2 to over 10 kb of ssDNA is generated depending on the kinetics of repair. Secondly, we determined the role of resection in the fidelity of DSB repair and gene targeting. Using mutants exhibiting decreased rates of resection we show that the length of 3′ tails defines the sequences involved in homology search and recombination donor choice. We demonstrate that normal resection at a DSB prevents deleterious repair via *de novo* telomere addition. Finally, we show that decreased resection was accompanied by dramatic increases in both the accuracy and efficiency of gene targeting. Together these results uncover a new role for resection in the regulation of DSB repair pathways.

## Results

### 3–6 kb of ssDNA accumulates at DSB ends during ectopic recombination

**Figure 1:**
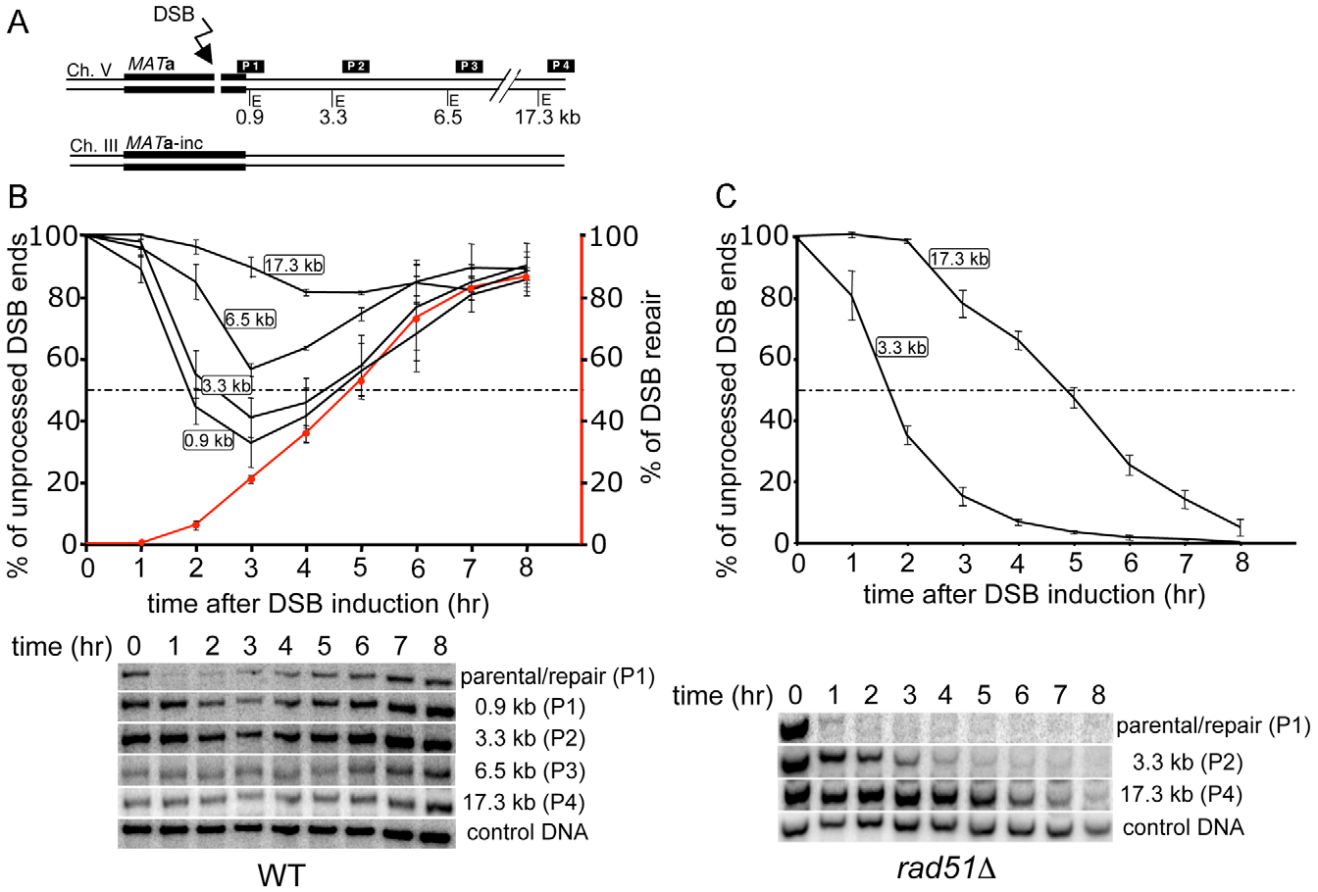
Measurement of resection in DSB–induced ectopic recombination. (A) Schematic representation of the ectopic recombination assay between *MAT* **a** on chromosome V and *MAT* **a**-inc on chromosome III. Positions of *Eco*RI sites (E) and DNA probes used for hybridization with respect to the HO recognition site on chromosome V are shown (tGI354). *MAT* **a** and *MAT* **a**-inc share 1.9 kb of homology in total. (B) Plot showing the percentage of unprocessed 5′ strand for each *Eco*RI site and the corresponding Southern blots are shown. Kinetics of DSB repair product formation is indicated by the red line. Plotted values are the mean values ±SD from three independent experiments. (C) 5′ strand processing was monitored and plotted from an ectopic recombination assay in *rad51*Δ cells (tGI379) as in (B).

Formation of ssDNA at DSB ends was extensively studied in multiple assays where the DSB was unrepairable. These assays were very useful in identifying enzymes involved in resection, however DSBs are normally repaired quickly by homologous recombination. Here for the first time we examined the kinetics and size of ssDNA generated at DSBs repaired by gene conversion and investigated the fidelity of DSB repair in mutants with limited resection. First, we measured ssDNA at a DSB repaired by an ectopic recombination assay in haploid cells where HO endonuclease induces a break within a *MAT* **a** sequence inserted at the *ARG5,6* locus on chromosome V (Figure 1A) [22]. This *MAT* **a** sequence shares 1.9 kb (1.4 kb proximal and 0.5 kb distal from the HO break) homology with *MAT* **a**-inc on chromosome III. *MAT* **a**-inc carries a point mutation in the HO recognition site that prevents HO cleavage. The DSB induced at *MAT* **a** locus is processed to form 3′ ssDNA tails that invade a homologous template *MAT* **a**-inc sequence located on chromosome III and initiate new DNA synthesis. Within one hour of HO nuclease induction a DSB occurred in all cells and the subsequent repair of the DSB takes about 3 to 7 hours in wild-type cells (Figure 1B) [22]. In this assay as well as in all other assays presented in this work, HO induction is continuous, therefore when the break is repaired by nonhomologous end joining, HO endonuclease cuts the *MAT* sequence again. We determined the amount of ssDNA generated during repair by following the temporary loss of several *Eco*RI restriction enzyme cleavage sites in the vicinity of the DSB as previously described (Zhu et al. 2008). Our goal was to find the distance from the HO cut site where at least half of the cell population removes the 5′ strand during repair. We used 4 probes that detect resection beyond 0.9 kb, 3.3 kb, 6.5 kb, and 17.3 kb from the break. As shown in Figure 1B, at least 70% of cells degraded DNA beyond 0.9 kb from the DSB ends and about half of the cell population degraded the 5′ strand beyond 3.3 kb from the DSB ends. Only 85% of cells repaired the break, therefore degradation of the 5′ strand beyond 17.3 kb from the HO cut site likely results from resection in cells that did not repair the break. Unrepaired breaks continue to be resected for at least 36 hours [[5] and data not shown]. The maximum amount of ssDNA at the DSB is observed at the time when the first recombination product starts to accumulate at 3 hours after HO endonuclease induction. To confirm that resection rate at the DSB induced on chromosome V is comparable to our previous estimation on chromosome III, we measured the resection in a strain lacking an essential protein for gene conversion, Rad51. As in wild-type cells, an HO break was induced in all cells within one hour (Figure 1C). Resection was measured 3.3 and 17.3 kb distal to the DSB in *rad51*Δ mutant cells (Figure 1C). The average rate of resection was 3.6 kb/hr, which is very similar to the rates determined in donorless wild-type and *rad51*Δ strains at the *MAT* locus on chromosome III [5]. A similar rate of resection was previously estimated at several loci on different chromosomes and on a plasmid substrate [20], [21], [23]. We concluded that the locus we used to measure resection during repair is representative and that half of the cell population resected at least 3–6 kb of the 5′ strand on one DSB end during repair. Given that 3′ ends are stable for several hours following break induction [20], [21] this is presumably the average amount of ssDNA that is active in the search for homologous sequences in this ectopic recombination assay. This amount of ssDNA is several times more than the homologous sequence available (0.5 kb). It is therefore probable that more ssDNA is formed than is required for repair. However this extensive resection may help to maintain DNA damage checkpoint arrest and give cells the necessary time for repair.

### The length of ssDNA at the DSBs depends on the kinetics of repair

**Figure 2:**
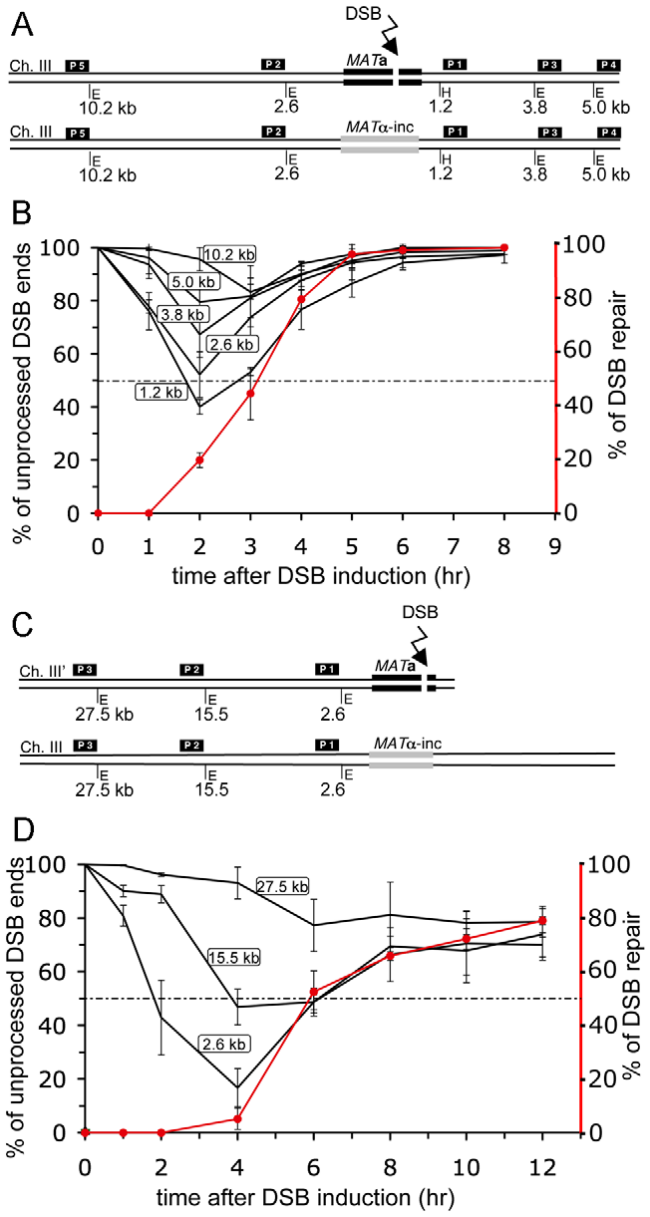
Kinetics of repair determine the amount of ssDNA during DSB repair. (A) Schematic representation of the allelic recombination assay between *MAT* **a** and *MAT*α-inc loci on chromosome III. Positions of *Eco*RI (E) and *Hin*dIII (H) sites and DNA probes used for Southern hybridization to analyze 5′ strand processing with respect to the HO recognition site are shown (yGI234). (B) Plot showing the percentage of unprocessed 5′ strand for each restriction site. Plotted values are the mean values ±SD from three independent experiments. Kinetics of DSB repair is indicated by the red line. (C) Schematic diagram of the BIR assay used for resection analysis. (D) 5′ strand processing was analyzed in the BIR repair strain (AM1003) as described in (B).

Normally DSB repair occurs with a fully homologous template molecule such as a sister chromatid or homologous chromosome. We therefore also estimated the amount of ssDNA generated during allelic recombination where the break is induced at the *MAT* **a** locus on chromosome III and is repaired by homologous recombination with a *MAT*α-inc sequence on a homologous chromosome III (Figure 2A). Allelic recombination is faster than ectopic as it is completed within 2 to 4 hours (Figure 2B and Figure S1). Faster kinetics of allelic recombination in diploid cells when compared to ectopic recombination in haploid cells is not due to *MAT* **a**/α heterozygosity. We previously demonstrated that ectopic recombination in *MAT* **a**/α heterozygous haploid cells and in *MAT* **a** haploid cells is equally slow [22]. Also resection rate in haploid and diploid cells is the same (Figure S2). The maximum amount of ssDNA during allelic recombination accumulates at 2 hours after break induction (Figure 2B). We measured resection beyond 1.2 kb, 2.6 kb, 3.8 kb, 5.0 and 10.2 kb from the DSB ends (Figure 2B). Southern blots for each probe used in this assay are shown in Figure S1. At least half of the cells resected DSB ends beyond 1.2 kb and 2.6 kb from the DSB and only 20% beyond 5.0 kb. These are significantly shorter sizes of ssDNA than in ectopic recombination. Interestingly, a small fraction of cells (10%) resected beyond 10.2 kb of ssDNA, suggesting that sometimes sequences far away from DSB ends can be active in the homology search even during fast allelic recombination. We think that during gene conversion most of the detected ssDNA is formed prior to strand invasion and is likely active in search for homologous sequence because new DNA synthesis and final product occurs 1.5–3 hours after DSB formation and only 20 minutes after strand invasion (Figure 1 and Figure 2; [24] and J. Haber, personal communication).

The third assay we used to measure resection was break-induced replication (BIR) where only one DSB end is homologous to the template DNA and, after strand invasion, the 3′ end is extended to copy the chromosome to its very end (Figure 2C) [25]. In this assay the break is induced at the *MAT* **a** locus, identical to the allelic recombination assay investigated above. It is a slow repair pathway as it takes about 6 to 10 hours to see the recombination product (Figure S3) [25]. The slow kinetics of initial DNA synthesis in BIR is not caused by terminal nonhomology, because BIR is equally slow even when the DSB end is perfectly homologous with its template [25].We measured 5′ strand resection in the BIR assay at 2.6 kb, 15.5 kb and 27.5 kb from the DSB end (Figure 2C). Southern blots for all probes used for this assay are shown in Figure S3. As shown in Figure 2D, most cells resected the 5′ strand beyond 2.6 kb and about half of the cell population resected DSB ends beyond 15.5 kb from the break. No significant resection was observed beyond 27.5 kb from the cut site. BIR is distinct from gene conversion because strand invasion in BIR monitored by Rad51 recruitment (ChIP) to template DNA, occurs with kinetics similar to gene conversion (Jain et. all, 2009) while new DNA synthesis is detectable only 2–3 hours after strand invasion. Therefore the very long ssDNA created during BIR suggests that resection continues after strand invasion but before new DNA synthesis. Altogether we conclude that the amount of ssDNA created during repair depends on the kinetics of repair and secondly that resection, at least in BIR assay, continues after strand invasion and probably helps to maintain DNA damage checkpoint arrest until new DNA synthesis and final repair product forms.

### Resection determines the sequences involved in homology search and donor choice

**Figure 3:**
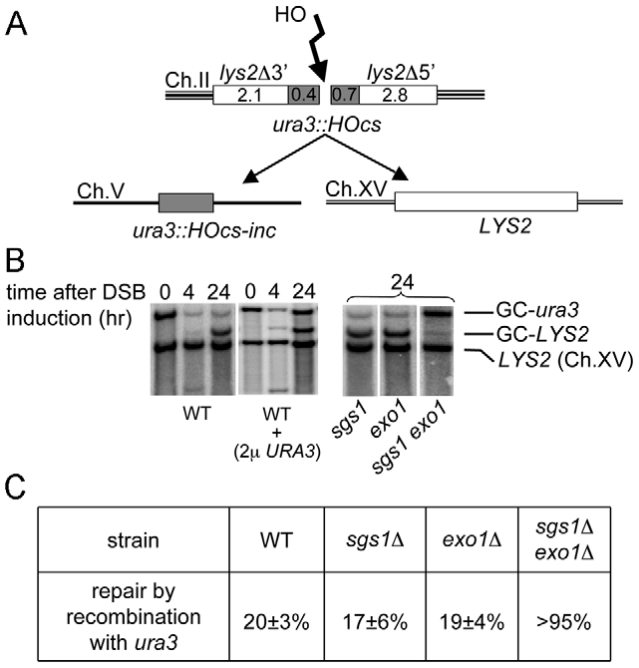
Resection at DSB ends determines the choice of recombination donor. (A) Schematic diagram of the competition assay used to determine whether sequences immediately next to the break (*ura3*) or sequences farther from the break (*lys2*) were used during gene conversion [30]. Details of the assay are described in the text. Sequence sizes are in kilobase pairs. (B) Southern blotting analysis of the competition gene conversion assay shown in (A). The *LYS2* gene was used as a probe. The positions of two gene conversion products called GC-*ura3* and GC-*LYS2* are indicated. (C) Frequencies of the choice of the *ura3::HOcs-inc* donor for DSB repair measured at 24 hr timepoint as the density of the band corresponding to the GC-*ura3* product divided by the combined density of both GC-*ura3* and GC-*LYS2* products in different mutants are shown in the table.

An implicit idea in current models of homologous recombination is that ssDNA determines sequences used for the homologous template search [26]. Now that we have verified the amount of ssDNA at DSBs, and the enzymes involved in resection are known, allowing us to manipulate the rate of ssDNA formation, we decided to verify this general concept. As shown above, an average of at least several kb of ssDNA is generated on one side of the break, which is much greater than the minimum homology required for efficient DSB repair. In DSB-induced recombination there is no difference in the efficiency of repair when homology is increased above 250 bp on each side of the break in a chromosomal context [27] and above 80 bp in a plasmid context [28]. Moreover, in yeast mating-type switching where an HO-induced break at the *MAT* locus is very efficiently repaired by recombination with the *HMR* and *HML* templates, the homology is limited to a few hundred base pairs [29]. Therefore, the length of ssDNA generated during resection at a DSB is greater than the minimum amount of sequence needed for efficient homology search and repair. It is possible that the formation of long ssDNA tails at DSBs increases the fidelity of repair by activating longer sequences in the homology search. Indeed, previously it was demonstrated that sequences located further away from the break are used for homology search preferentially over sequences within the first 0.5 kb from the break [30]. To verify whether resection determines the sequences involved in homology search and impacts template sequence choice we used a competition assay designed by Inbar and Kupiec [30]. In this assay, an HO break is induced on chromosome II at a *ura3* sequence that was inserted in the middle of the *LYS2* gene. A DSB at this site can be repaired by recombination with one of two homologous template sequences, *URA3* located on chromosome V or *LYS2* located on chromosome XV (Figure 3A). The total homology between *ura3* sequences is 1.1 kb and between *lys2* sequences is 4.9 kb. In agreement with the original report [30] we observed that *lys2* sequences that are further from the break are used preferentially as a recombination template. Only about 20±3% of wild-type cells repair the DSB by recombination with the *URA3* donor sequence. Given that during ectopic recombination an average of 3–6 kb of ssDNA accumulates at both sides of a DSB (Figure 1B), it is likely that *LYS2* sequences are used 4 times more often than *URA3* simply because they are 4 times longer. With the average rate of resection of 4 kb/hr, *ura3* sequences are resected within 7–8 minutes and *lys2* sequences are completely resected within an additional 30–40 minutes after DSB formation. When *URA3* sequences were increased to match the *LYS2* length, both templates were used with almost equal frequency [30]. Here we tested whether additional copies of *URA3* on centromeric or multicopy plasmids could influence the donor choice. As previously demonstrated, plasmid DNA can be efficiently used for repair of chromosomal DSBs [28]. When an additional *URA3* sequence was provided on a centromeric plasmid we did not observe any change in donor choice (data not shown). However, the presence of a multicopy plasmid (2μ) carrying *URA3* sequences increased *URA3* sequence usage for DSB repair to 55% (Figure 3B). This result suggests that sequences further away from the break are used often as a template for repair even if short sequences close to the break have multiple copies of intact templates. Another conclusion is that long Rad51 nucleofilaments have a higher chance of identifying single homologous sequence than short nucleofilaments even when multiple homologous sequences are available. This feature of homology search may inhibit recombination between short repeats. Finally, when both processive resection pathways dependent on Sgs1 and Exo1 were eliminated, *URA3* was almost the only donor used for repair (>95%) demonstrating that the ssDNA exposed during resection determines the sequences involved in homology search (Figure 3B and 3C). The viability of wild-type cells and of *sgs1*Δ or *exo1*Δ single mutants was comparable, while in the double mutant *sgs1*Δ *exo1*Δ, repair by gene conversion and viability are decreased to ∼11%. Altogether our data suggest that resection activates long stretches of ssDNA in the homology search and plays an important role in the choice of donor sequences.

### *de novo* telomere addition is a frequent pathway of DSB repair in resection-deficient *sgs1* **Δ** *exo1* **Δ** mutants

**Figure 4:**
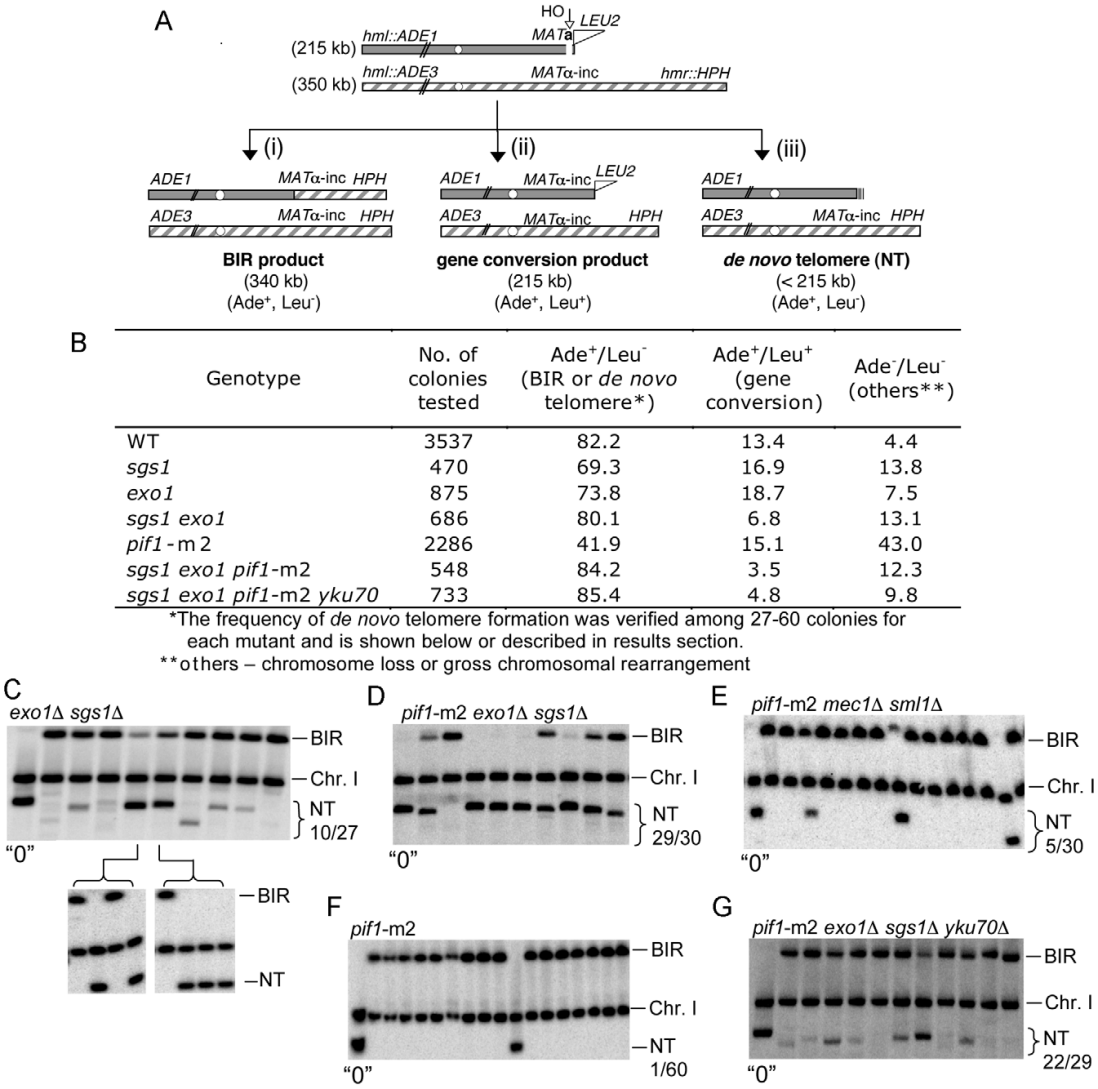
*De novo* telomeres are frequently formed at poorly processed DSB ends. (A) Schematic diagram showing chromosome structure of the disomic strain (AM1003) that carries an original chromosome III (350 kb) and a fragmented chromosome III (215 kb). Sequences distal to the HO recognition site were replaced by the *LEU2* gene and telomere sequences as described [25]. Three major pathways of repair with their respective product sizes are shown: BIR, gene conversion and *de novo* telomere addition (NT). Products can be distinguished by markers and/or by sizes on PFGE gels. (B) Frequency of DSB repair by BIR and gene conversion in the AM1003 strain and its derivatives is shown in the table. (C) PFGE analysis of products from Ade^+^ Leu^−^ colonies *exo1*Δ *sgs1*Δ, (D) *pif1*-m2 *exo1*Δ *sgs1*Δ, (E) *pif1*-m2 *med1*Δ *sml1*Δ (F) *pif1*-m2 and (G) *pif1*-m2 *exo1*Δ *sgs1*Δ *yku70*Δ mutant strains. To separate BIR repair products and telomere-added products (NT) in the *exo1*Δ *sgs1*Δ strain (C) originating from one survivor we streaked single colonies on YEPD plates and analyzed products from individual colonies by subsequent PFGE. Genomic DNA of products repaired by *de novo* telomere addition was purified and used for sequencing to identify telomere addition sites [(C,D) and Figure 5]. “0” indicates control with no break induction. Position of chromosome I (Chr.I) that carries *ade1–1* allele and is detected with ADE1 probe is shown.

**Figure 5:**
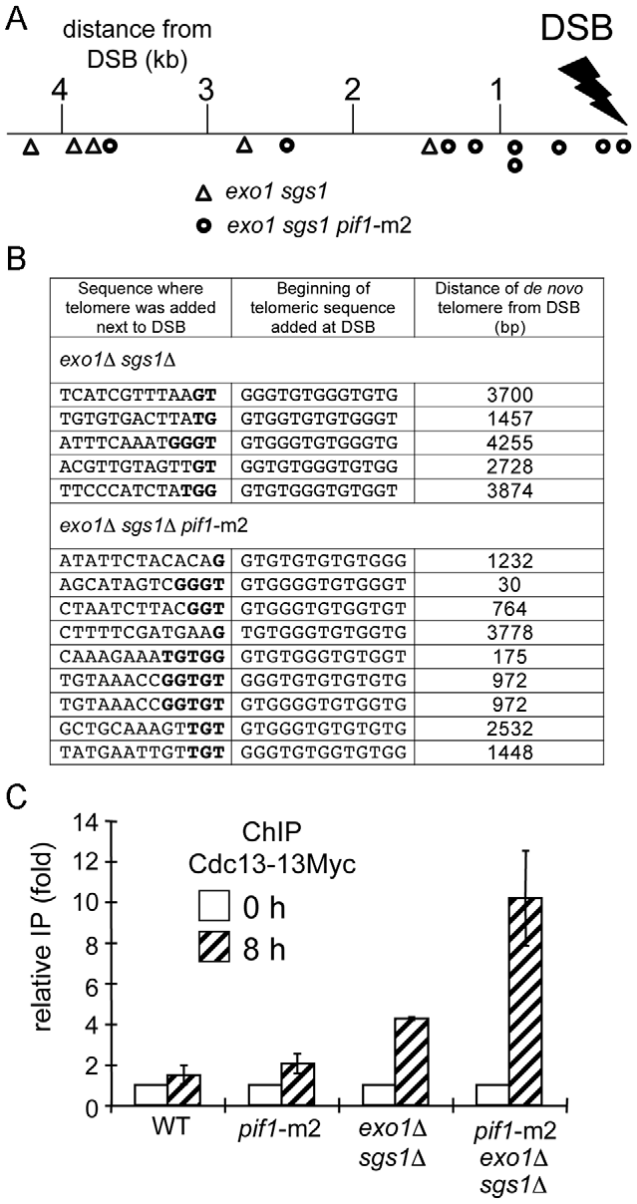
Analysis of telomere formation in *exo1*Δ *sgs1*Δ mutants. (A) The positions of new telomere addition in 14 products from *exo1*Δ *sgs1*Δ (5 triangles) and *pif1*-m2 *exo1*Δ *sgs1*Δ (9 circles) cells were determined by sequencing. (B) Exact position and sequence where telomeres were added is presented in the table. (C) ChIP analysis of Cdc13-13Myc binding to the region flanking the DSB was conducted in wild-type, *pif1*-m2, *exo1*Δ *sgs1*Δ and *pif1*-m2 *exo1*Δ *sgs1*Δ strains using polyclonal anti-myc antiserum. Samples were collected before and 8 h after DSB induction. Immunoprecipitated DNAs were amplified by qPCR using primer pairs to amplify a region located about 1 kb away from the HO break site. The 8 h time point IP values were normalized to the time 0 samples to yield the fold-IP values plotted on the Y-axis for the wild-type and each mutant strain. Error bars represent one standard error of the mean for three independent experiments.

We previously found that decreased resection in *sgs1*Δ *exo1*Δ cells leads to a partial deficiency in homologous recombination. In haploid cells it is not possible to follow the fate of broken unrepaired chromosomes because these cells die due to the loss of essential genes upon resection and missegregation of the acentric part of the chromosome. To verify the fate of broken chromosomes where the breaks are very poorly resected, we used a disomic strain that carries a second truncated copy of chromosome III with the HO break site close to its end (Figure 4A). In this assay most wild-type cells repair the break by BIR (82%), a small fraction of cells can repair the break by gene conversion (13%) using very short homology (46 bp) distal to the DSB, and the frequency of chromosome loss is only 4% (Figure 4B). These cells survive normally even when the DSB is not repaired because there is second copy of chromosome III that is not cut. The efficiency of repair was estimated by plating wild-type, *sgs1*Δ, *exo1*Δ and *sgs1*Δ *exo1*Δ cells on YEPGal plates and replica-plating the grown colonies onto plates lacking adenine or leucine. In *exo1*Δ and *sgs1*Δ single mutants, there was a slight increase of chromosome loss and gene conversion at the expense of BIR consistent with a previous report (Figure 4B) [31]. In *sgs1*Δ *exo1*Δ cells, DSB repair efficiency unexpectedly is comparable to wild-type cells and most cells maintain the chromosome that was cut by HO endonuclease. Importantly, Ade^+^ colonies are not sectored, indicating that the chromosome that was cut by HO endonuclease is maintained well for generations. Previous estimations of DSB repair by gene conversion in *sgs1*Δ *exo1*Δ mutant haploid cells showed a decrease by half or more [4], [5]. To make sure that the Ade^+^ Leu^−^ colonies correspond to repair by BIR, we verified the size of 27 individual repair products from Ade^+^ Leu^−^ colonies using pulsed-field gel electrophoresis (PFGE) and subsequent probing with an ADE1 probe. We observed that all cells have products corresponding to the expected BIR repair product size, however 10 out of 27 have an additional product or several products similar in size to the initial chromosome III or slightly shorter. Examples of these products are shown in Figure 4C. Short chromosome products correspond to 9% of total products from 27 Ade^+^ Leu^−^ colonies as measured by the relative band intensities of all the repair products. The presence of two or more products, often observed as bands with different intensities, suggested that the repair process occurred after cells divided or that the two sister chromatids were repaired by different pathways. As previously demonstrated, *sgs1*Δ *exo1*Δ cells are deficient in the damage checkpoint response [3], [5], and because DSB ends are not processed normally in these cells, a broken chromosome is not lost but instead cells divide with the damaged chromosome that eventually is repaired. To check whether these repair products are within the same cell or in independent cells we streaked several Ade^+^ Leu^−^ colonies for single cells and further analyzed five such newly grown individual colonies for their repair products. In this case only either a BIR product or a single short product was observed (Figure 4C). The alternative products are very similar in size to the cut chromosome III, suggesting that they arose by *de novo* telomere formation. To verify this, we sequenced short chromosome ends and confirmed new telomere formation mostly within the first 5 kb from the DSB (Figure 5A and 5B). Rarely telomeres were added at a distance of over 10 kb away from the break, suggesting that even in *sgs1*Δ *exo1*Δ cells the broken chromosome is eventually degraded and telomeres are added far from the HO break site. The 3′ ends are stable only for several hours and later are degraded [20]. Therefore, it is likely that both strands of unprotected ends are eventually degraded in *sgs1*Δ *exo1*Δ cells and telomeres can be added further away from the break. Examples of sequences where *de novo* telomeres were formed are shown in Figure 5A and 5B. Most telomeres were added at short 1–5 GT-rich sequences, a characteristic similar to spontaneous telomere formation [32]. Therefore, slow resection greatly stimulates *de novo* telomere formation, but does not change the sequence preference where telomeres are added. Importantly, we verified 30 Ade^+^ Leu^−^ colonies in *sgs1*Δ *exo1*Δ single mutant and in wild-type cells and did not observe any short *de novo* telomere products (data not shown) suggesting that these enzymes have a redundant function in inhibiting *de novo* telomere formation at DSBs likely related to their role in resection.

### New telomere formation during allelic recombination in *sgs1*Δ *exo1*Δ cells

In the BIR assay we described above, the HO break is close to a chromosome end that could stimulate telomere formation in cells with poor resection. To test whether proximity to telomeres affects new telomere formation in resection deficient cells, we used a diploid *sgs1*Δ/*sgs1*Δ *exo1*Δ/*exo1*Δ strain to carry out an allelic recombination assay between two chromosomes III identical to the one used for resection verification (Figure 2A). Here the break is induced at the *MAT* **a** locus in the middle of the chromosome. The proximal and distal parts of the chromosome that is cut by HO endonuclease has *ADE1* and *THR4* markers, respectively, that allowed us to follow product formation on minimal media plates (Figure S4). 19% of cells repair the break by gene conversion (Ade^+^ Thr^+^) and only 2% show chromosome loss (Ade^−^ Thr^−^). The lower level of gene conversion in this assay compared to the ectopic recombination assay between *MAT* **a** and *MAT* **a**-inc [5] likely results from the necessity for resection of a 0.7 kb long Y**a** sequence during allelic recombination between *MAT* **a** and *MAT*α-inc. The rest of the cells maintained the *ADE1*-marked centromeric part of the chromosome that could correspond to either BIR or *de novo* telomere addition. To distinguish between these repair pathways, we analyzed products from 29 Ade^+^ Thr^−^ colonies by PFGE and again observed a dramatic increase in new telomere formation at the DSB sites. All tested Ade^+^ Thr^−^ colonies contained BIR products and 6 out of these (∼20%) also contained *de novo* telomere products (Figure S4). Therefore, slow resection stimulates *de novo* telomere formation even when the break is in the middle of the chromosome. In a similar study at the same locus in *rad52*Δ mutant cells that simply cannot repair a DSB but resect normally, *de novo* telomeres are not formed unless long telomere seeding sequences are provided [33]. Altogether these data suggest that slowly processed DSB ends gain features that predispose them to form *de novo* telomeres.

### Resection and Pif1 independently suppress *de novo* telomere formation at DSBs

Pif1 is a major telomerase inhibitor in budding yeast [34]. In *pif1*Δ mutants, telomeres are longer and new telomeres are formed more frequently at spontaneous and induced chromosomal breaks [[35], [36], [37]; reviewed in [38]]. Interestingly, Pif1 was shown recently to be phosphorylated upon DNA damage in a Mec1-Rad53-Dun1-dependent manner and this phosphorylation is needed for Pif1 to inhibit *de novo* telomere formation [39]. Previously, we and others demonstrated that during G2/M DNA damage checkpoint arrest, Mec1/Ddc2 recruitment and Rad53 phosphorylation are partially defective in *sgs1*Δ *exo1*Δ cells [3],[5]. Therefore, we thought that increased *de novo* telomere formation in *sgs1*Δ *exo1*Δ mutant cells could be due to poor damage checkpoint activation and lack of phosphorylated Pif1 [39]. To explore this possibility, we tested whether the frequent *de novo* telomere formation phenotypes observed in both *sgs1*Δ *exo1*Δ and *pif1*Δ mutants are epistatic. We examined DSB repair products in *pif1*-m2 and *pif1-*m2 *sgs1*Δ *exo1*Δ mutant cells. The *pif1*-m2 mutation eliminates the nuclear form of Pif1 while mitochondrial Pif1 is still present [37]. Surprisingly, we found that *pif1*-m2 single mutants are defective in BIR, as only half of the colonies retain growth on ade^-^ plates (Figure 4B). The detailed analysis of Pif1's role in BIR will be presented elsewhere (W.H.C. and G.I., data not shown). Here we analyzed repair products from 30 individual Ade^+^ Leu^−^ colonies of *pif1-*m2 *sgs1*Δ *exo1*Δ triple mutant cells and 60 individual Ade^+^ Leu^−^ colonies of *pif1-*m2 mutant cells (Figure 4D and 4F, and data not shown). Only 1 out of 60 Ade^+^ Leu^−^ products from *pif1*-m2 cells showed repair by *de novo* telomere formation (Figure 4F). However, in *pif1-*m2 *sgs1*Δ *exo1*Δ triple mutants almost all (29/30) products contained either BIR and *de novo* telomere or just *de novo* telomere products (Figure 4D). *De novo* telomere products constituted 53% of all products from Ade^+^ Leu^−^ colonies as measured by the intensity of the bands corresponding to the BIR and *de novo* telomere products. In *pif1-*m2 *sgs1*Δ *exo1*Δ triple mutants, we observed much better maintenance of the broken chromosome when compared to *pif1*-m2 cells (Figure 4B), however this increase in Ade^+^ colonies turned out to correspond to repair via new telomere addition. Similar results we obtained in *pif1-4A sgs1*Δ *exo1*Δ mutant where pif1 phosphorylation mutant protein was defective in inhibiting new telomere formation at DNA breaks [39]. In *pif1-4A sgs1*Δ *exo1*Δ mutant 19 out of 20 products from Ade^+^ Leu^−^ colonies formed new telomeres (data not shown). In conclusion these results demonstrate that Pif1 actively suppresses new telomere formation even in cells that are partially defective in the DNA damage checkpoint response. We sequenced the ends of *de novo* telomeres in *pif1-*m2 *sgs1*Δ *exo1*Δ mutant cells and showed that most of them are formed within the first 1 kb from the DSB, which are even closer than those observed in *sgs1*Δ *exo1*Δ double mutant cells (Figure 5A and 5B). In triple *pif1-*m2 *sgs1*Δ *exo1*Δ mutants, there is no further decrease in resection when compared to *sgs1*Δ *exo1*Δ cells (Figure S5), indicating that Sgs1/Exo1 and Pif1 independently and in different way suppress *de novo* telomere formation at DSB ends.

### Analysis of new telomere formation at DSBs in checkpoint deficient cells

In *sgs1*Δ *exo1*Δ mutant cells, checkpoint response in response to a single DSB is decreased [3], [5]. Therefore one possible reason for the very high rate of new telomere formation observed in *sgs1*Δ *exo1*Δ cells is the decreased damage checkpoint response. However we clearly demonstrated in the section above that Pif1, which needs to be phosphorylated by Mec1 in order to inhibit new telomere formation, is still active in *sgs1*Δ *exo1*Δ cells. To examine further whether decreased checkpoint response in *sgs1*Δ *exo1*Δ cells is exclusively responsible for the very high level of new telomere formation, we tested DSB repair (BIR assay) in *rad24*Δ and *rad9*Δ mutants that are damage checkpoint deficient. Again we analyzed 20 products from Ade^+^ Leu^−^ colonies from each mutant by PFGE. We found only 1 out 40 products in *rad24*Δ corresponds to new telomere formation (data not shown). Further we constructed a triple mutant *mec1*Δ *sml1*Δ *pif1-*m2 and again analyzed the frequency of new telomere formation. We found only 5 out of 30 products correspond to new telomere formation (Figure 4E), which is significantly more than in *pif1*-m2 but also much less than in *sgs1*Δ *exo1*Δ *pif1*-m2 where almost all Ade^+^ Leu^−^ colonies contained new telomere products. Together these data suggest that checkpoint deficiency in *sgs1*Δ *exo1*Δ cells alone does not explain the very high rate of new telomere formation, and points towards resection as an important factor.

### Slow resection stimulates recruitment of Cdc13 and partially bypasses the Ku complex requirement for *de novo* telomere formation

Telomerase is recruited to single-stranded overhangs at the ends of chromosomes via Cdc13 and the Ku complex [reviewed in [40]]. Both proteins are needed for *de novo* telomere formation at DSBs [36], [41]. Deletion of *YKU70/80* almost completely suppresses the increased *de novo* telomere formation observed in *pif1*Δ cells [36]. Another function of Ku at telomeres is protection from nucleases [42], [43], [44]. In *sgs1*Δ *exo1*Δ cells, DSB ends are processed minimally and so the function of the Ku complex is presumably limited to telomerase recruitment. To test whether slow resection at DSBs bypasses the need for Ku in *de novo* telomere formation, we constructed the quadruple mutant *pif1-*m2 *sgs1*Δ *exo1*Δ *yku70*Δ and measured the frequency of *de novo* telomere formation at DSB ends. As shown in Figure 4G, most of the DSBs still lead to *de novo* telomere formation (22/29), however the total intensity of new telomere products dropped to 15% compared to 53% observed in *pif1-*m2 *sgs1*Δ *exo1*Δ cells. Similarly in *sgs1*Δ *exo1*Δ *yku70*Δ cells we observed a relatively high number of new telomere formation (10 out of 40 products) comparable to *sgs1*Δ *exo1*Δ. However, the intensity of new telomere product was again reduced (data not shown). Therefore, slow resection exhibited by *sgs1*Δ *exo1*Δ mutants partially suppresses the need for the Ku complex in *de novo* telomere addition at DSBs. The Ku complex plays the opposite role of Sgs1 and Exo1 as it protects DNA ends from degradation. Because in the absence of resecting enzymes Ku becomes partially dispensable for new telomere formation, we propose that the role of Sgs1 and Exo1 specifically in resection, rather than other functions of these enzymes, is important in preventing new telomere formation at DSBs.

Cdc13 was shown to be recruited to a DSB in the middle of a chromosome even in wild-type cells where telomeres are not formed [45]. To verify whether Cdc13 recruitment is stimulated at poorly resected DSB ends we performed chromatin immunoprecipitation (ChIP) with Myc-tagged Cdc13 in wild-type, *pif1*-m2, *sgs1*Δ *exo1*Δ and *pif1-*m2 *sgs1*Δ *exo1*Δ mutant cells. We measured Cdc13 recruitment before HO break induction and 8 hours after break induction when significant BIR products start to accumulate [25]. As shown in Figure 5C, recruitment of Cdc13 increased at 8 hr after break induction in *sgs1*Δ *exo1*Δ and *pif1*-m2 *sgs1*Δ *exo1*Δ cells about 3- to 6-fold relative to wild-type cells. These results suggest that slow resection results in higher or longer lasting recruitment of Cdc13 that may then stimulate *de novo* telomere formation.

### Gene targeting efficiency and accuracy increase dramatically in *sgs1*Δ *exo1*Δ cells

**Figure 6:**
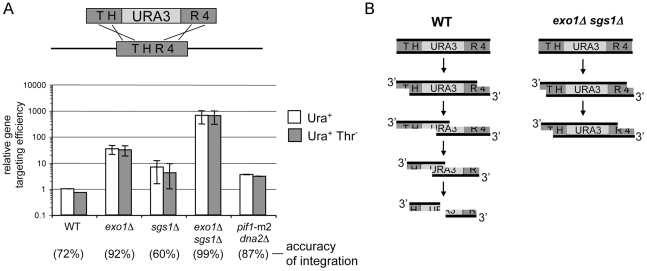
Elimination of enzymes that degrade DNA ends greatly stimulates gene targeting. (A) A 2.1 kb *thr4::URA3* cassette was used for a gene replacement assay in wild-type and mutant strains lacking one or more enzymes involved in DSB resection. Efficiency and accuracy of gene targeting measured as the amount of Ura^+^ or Ura^+^ Thr^−^ colonies is displayed. Error bars represent standard deviation for three independent experiments. (B) Hypothetical model presenting gene targeting in cells exhibiting normal processing of DNA ends and in cells with decreased resection. Extensive resection exposes the nonhomologous *URA3* marker sequence and eventually degrades the transformed cassette. Exposed 3′ homologous tails are likely to be degraded faster in wild-type cells than in *sgs1*Δ *exo1*Δ mutant cells.

While constructing additional gene deletions in *sgs1*Δ *exo1*Δ strain we observed that gene targeting in *sgs1*Δ *exo1*Δ mutants was surprisingly very efficient. This is in contrast to a general decrease in DSB-induced recombination which we tested in the different assays described above. We attempted to delete several genes in *sgs1*Δ *exo1*Δ cells and in each case gene targeting was highly efficient. For example, deletion of *ygr042w* ORF with *ygr042w*::KanMX cassette was 140-fold higher in *sgs1*Δ *exo1*Δ than in wild-type cells. To examine more carefully the efficiency of gene targeting we transformed a *thr4*::*URA3* cassette containing 1.1 *Sma*I-*Hin*dIII *URA3* fragment into *ura3–52* wild-type cells, and derivative *sgs1*Δ, *exo1*Δ, *dna2*Δ *pif1*-m2 and *sgs1*Δ *exo1*Δ mutant cells and determined the amount of Ura^+^ and Ura^+^ Thr^−^ colonies. We further normalized the level of Ura^+^ colonies to the plating efficiency of each strain and compared the efficiency of gene targeting in wild-type and mutant cells. As shown in Figure 6A, the targeting efficiency increased in all single mutants, by 3- to 4-fold in the absence of Sgs1 or Dna2 and over 30-fold in the absence of Exo1, relative to wild-type cells. Strikingly, when both Sgs1 and Exo1 are absent the efficiency of targeting increases over 600-fold. It is likely that in the absence of enzymes which process DSB ends the stability of transformed DNA is increased and therefore gene targeting is more efficient. We also measured the accuracy of targeting among over 200 Ura^+^ colonies and observed that in *sgs1*Δ *exo1*Δ mutant cells almost all targeting events are accurate (99%), which is significantly higher than in wild-type cells (72.2%; p<0.05). It is possible that in wild-type cells quick degradation of the 5′ strands exposes the *URA3* marker sequence of the targeting cassette and activates it in a homology search that leads to integration at a locus other than *THR4*. The strain we transformed was *ura3–52*, therefore one possibility was that the *thr4::* *URA3* cassette was integrated at *ura3–52* locus. To test this possibility we transformed the same *thr4::* *URA3* cassette into the strain where the entire *URA3* ORF was deleted [46]. In this strain, the *URA3* marker within the cassette shared no homology with the genome. However, again we observed that only 78% of gene targeting was accurate. Therefore inaccurate integration is not caused by resection reaching *URA3* sequences within cassette that stimulates integration at the *ura3–52* locus. However it is still possible that marker sequence within the cassette can, when resected, inhibit accurate integration. The accuracy of gene targeting rose from 72% to 87% or 95% when we increased the flanking homology length within *thr4::URA3* cassette from 1.1 to 2.5 or 6.5 kb. This suggests again that cells use longer stretch of ssDNA in homology search and it is beneficial for the repair accuracy. With longer homology we also observed 6–7 fold increase in targeting efficiency in wild-type cells, which is still 2 orders of magnitude less than the increase observed in *sgs1*Δ *exo1*Δ cells. Only a slight increase in efficiency of targeting with longer homology but normal resection is not surprising because the resection rate (4 kb/hr) is high enough to degrade the cassette very quickly even when flanking homology is relatively long.

## Discussion

### Benefits of extensive resection during DSB repair: fidelity of repair and checkpoint maintenance

We determined for the first time the length of ssDNA generated during DSB repair and showed that resection determines sequences used for homology search. Three different assays with different kinetics were used to measure resection: allelic recombination, ectopic recombination and BIR. Allelic recombination lasts about 2–3 hours which is similar to recombination between sister chromatids [47] and the average length of ssDNA accumulated during such repair is only 2–4 kb on each side of the break. Longer ssDNA (3–6 kb) was formed during the slower ectopic recombination assay. In these two assays, most of ssDNA accumulated before strand invasion, as the final repair product is formed 1.5 to 3 hours after DSB induction and only 20 minutes after strand invasion (Figure 1 and Figure 2; [24] and J. Haber, personal communication). We suggest that most ssDNA we detected in gene conversion assays participates in homology search. The 2–6 kb of ssDNA greatly exceed the amount of homology needed for efficient repair but may be beneficial for the fidelity of repair. If only very short sequences next to the break are involved in the homology search it may lead to recombination with short repeats. Involvement of longer ssDNA tails in the homology search would limit such recombination events. Indeed we demonstrated that in a mutant that generates only very short ssDNA at DSB ends, the homology search is limited to the vicinity of the break and repair involves only a short repeated sequence. In genomes with high numbers of short repeats, such as the 1 million copies of the 0.3 kb-long Alu repeats found in the human genome, involvement of longer sequences in the homology search can constitute an additional barrier for nonallelic recombination besides mismatch repair or the cohesin-mediated bias toward intersister chromatid recombination. However, resection is a double-edged sword as activation of longer ssDNA in the homology search also brings the risk of involvement of repetitive sequences located further away from the break [48]. Equal involvement of both DSB ends probably decreases such events [49]. Also, cells are able to downregulate resection to prevent too extensive chromosome degradation at DSBs [13].

Another reason why cells resect long ssDNA during repair probably relates to DNA damage checkpoint activation and maintenance. This could be particularly important during very slow repair by BIR where we found 10–15 kb of ssDNA. In BIR strand invasion occurs with kinetics comparable to gene conversion but 3′ ends prime new DNA synthesis only 2–3 hours later [25], [49]. The much longer ssDNA formed during BIR than during gene conversion suggests that resection continues after strand invasion. It is likely that this extensive resection after heteroduplex formation stimulates maintenance of DNA damage checkpoint arrest until cells repair the break. In conclusion short ssDNA might be sufficient for strand exchange processes but not for the checkpoint arrest that is needed to complete repair.

### *De novo* telomere formation at poorly resected DSB ends

Telomere addition at spontaneous or induced DSBs was previously described in yeast, mouse and human cells [reviewed in [38]]. It is an extremely rare chromosome aberration in wild-type cells but frequent in tumor cells. Here we observed that cells in which resection is limited to a few hundred base pairs frequently use the alternative pathway of repair via *de novo* telomere addition. When the yeast inhibitor of telomerase, Pif1, was eliminated in poorly resecting mutants, most cells repaired the DSB using *de novo* telomere addition. Why do *sgs1*Δ *exo1*Δ mutant cells very frequently use the alternative pathway of repair via *de novo* telomere addition? We think that a combination of repair, checkpoint and resection defect in this mutant is a key for the high level of new telomere addition at DSBs. Separately repair or checkpoint defective mutants do not affect new telomere formation so dramatically. In mutants such as *rad52*Δ that do not repair the breaks, new telomeres are not added [33] or added infrequently [35]. Also in checkpoint deficient cells like *rad24*Δ or *rad9*Δ there is only a slight increase in new telomere formation at DSBs. However in a *sgs1*Δ *exo1*Δ mutant, besides repair and checkpoint deficiency, decreased degradation at a DSB site prevents chromosome loss that gives cells more chance for alternative repair. Indeed all *de novo* telomeres in *sgs1*Δ *exo1*Δ and more than half in *pif1*-m2 *sgs1*Δ *exo1*Δ mutants formed only when cells divided once or more times after DSB induction. Another factor stimulating *de novo* telomere formation is that in *sgs1*Δ *exo1*Δ cells binding of Cdc13, a protein essential for telomerase recruitment, is increased. It is possible that at poorly resected DSBs like at telomeres that have very short ssDNA overhangs, Cdc13 can successfully compete with RPA for ssDNA. We also demonstrated that decreased resection partially bypassed the need for Ku70/80 for *de novo* telomere formation. The Ku complex is required for spontaneous and MMS-induced *de novo* telomere addition in cells with normal resection [36], [41]. Ku contributes to this process by direct recruitment of telomerase and/or by protecting DSB ends from degradation. Here we demonstrate, in a strain where ends are relatively stable with minimal ssDNA formation, a dramatic increase in *de novo* telomere formation even in the absence of Ku. The fact that deletion of both Sgs1 and Exo1 partially bypasses the need for the Ku complex that protects DNA ends from degradation indicates that the role of Sgs1 and Exo1 in resection, rather than another function of these enzymes, is important in preventing new telomere formation at DSBs.

### Gene targeting in the absence of enzymes that degrade ssDNA

Gene targeting is a major technique in molecular biology that allows the precise modification of the genome and is envisaged as being a major future therapeutic approach for genetic disorders. One of the major problems of gene targeting in higher eukaryotes is the low efficiency and particularly the very low accuracy of this process. Here we observed a dramatic increase in gene targeting efficiency and accuracy in the absence of enzymes which resect 5′ strands, Sgs1 and Exo1 (Figure 6A). In the absence of both Sgs1 and Exo1, DSB ends are processed by the MRX complex and Sae2 to generate 100–1,000 bp of ssDNA [4], [5]. This is similar in size to the homologous sequence frequently provided as transformed DNA for gene targeting. We suggest that transformed DNA is similarly processed initially by the MRX complex and later by Exo1 and Sgs1. In the absence of both Sgs1 and Exo1, this transformed targeting cassette is probably stable for much longer than in wild-type cells, having only short ssDNA at the ends active in the homology search (Figure 6B). Together this gives a higher chance for correct integration into the genome. Longer homology within the transformed cassette increases gene targeting only slightly (6–7 fold), and is not comparable to the increase observed in the absence of processive resection enzymes. This is not surprising given the high 4 kb/hr resection rate. Sgs1 has an additional role in DSB repair as it suppresses the crossover pathway, presumably by double Holliday junction dissolution [22], [50]. Gene targeting presumably relies on crossover events between the transformed DNA cassette and the chromosome, therefore it is possible that besides decreased resection the absence of Sgs1 can stimulate targeting by increasing the crossover pathway. In the absence of both Sgs1 and Exo1 the accuracy of gene targeting is also increased. One possibility is that exposure of marker sequences within the transformed cassette inhibits correct integration even when the marker has no homology within the genome. Future experiments with cassettes that do not have a marker will help to investigate this possibility. An alternative explanation could be that quick exposure of the 3′ ends may lead to their degradation [20] leaving less homology on the ends of the transformed cassette and therefore more inaccurate targeting. It is likely that in human cells where Exo1 and BLM play comparable roles in resection to their yeast counterparts [3], gene targeting efficiency or accuracy could be increased by temporary depletion of both enzymes. Indeed, in human cells depletion of BLM helicase stimulates gene targeting [51]. However, in human cells there are additional nucleases that are not present in yeast such as cytoplasmic/nuclear TREX1 3′ exonuclease that is expressed in all cells. TREX1 was shown to degrade DNA arising from DNA replication, DNA damage, endogenous retroviruses or viral infection and *TREX1*-null mice accumulate DNA leading to chronic damage checkpoint activation [52], [53] [54]. These phenotypes strongly suggest that transformed DNA cassettes are a substrate for TREX1 as well. We think that one reason for the relatively less efficient and much less accurate gene targeting observed in human and other organisms when compared to yeast is due to the presence of additional nucleases that degrade transformed DNA. Future experiments will examine whether TREX1 interferes with gene targeting. A tempting speculation is that Sgs1 and Exo1 degrade any linear DNA in cells like the cDNA of retrotransposons or viral DNA thus protecting the genome from integration of foreign DNA. Accordingly in the absence of Sgs1, the frequency of retrotransposition increases in yeast [55].

Taking these observations together we have demonstrated that the extent of resection from a DSB has a fundamental role in determining the outcome of DSB repair and in the maintenance of genome stability. It will be important to understand how resection is regulated at a molecular level by checkpoint proteins and by chromatin remodeling to diminish the deleterious pathways of repair.

## Materials and Methods

### Strains

All strains used here are derivatives of four strains: (i) tGI354 to study ectopic recombination (*hml::* *ADE1 MAT* **a** *-inc hmr::ADE1 ade1 leu2–3,112 lys5 trp1::hisG ura3–52 ade3::GAL::HO arg5,6::HPH::MAT* **a**); (ii) MK181, a gift from Martin Kupiec, to study recombination template choice with respect to resection distance (*MAT* **a**-*inc ura3-HOcs-inc ade3::GAL-HO ade2-1 leu2–3,112 his3–11,15 trp1–1 can1–100*); (iii) yGI234 to study allelic recombination (*hml::ADE1/HML MAT* **a** */MAT*α-inc *hmr::ADE1/HMR ade1/ade1 leu2–3,112/leu2–3,112 lys5/LYS5 trp1/trp1 ura3–52/ura3–52 THR4/thr4::URA3 ade3::GAL::HO/ADE3)*; and (iv) disomic AM1003 to study BIR (*MAT* **a** *-LEU2-tel/MAT*α-inc *ade1 met13 ura3 leu2–3,112/leu2 thr4 hml::ADE1/hml::ADE3 hmr::HPH ade3::GAL-HO* FS2::NAT/FS2). A list of all strains is presented as supplemental material (Table S1).

### Measurement of 5′ strand resection and DSB repair

Measurement of resection during DSB repair was done by following the transient loss of restriction enzyme cutting sites at different distances from the break. DNA isolated by glass bead disruption using a standard phenol extraction method was digested with restriction enzymes and separated on 0.8% agarose gels. Southern blotting and hybridization with radiolabeled DNA probes was carried out as described previously [56]. Multiple DNA probes used for hybridization to detect 5′ strand resection beyond the restriction site, as well as the sequences of DNA primers used to prepare the probes by PCR, are listed in Table S2. Intensities of bands on Southern blots corresponding to probed DNA fragments were analyzed with ImageQuant TL (Amersham Biosciences). Quantities of DNA loaded on gels for each time point were normalized using a *TRA1* gene DNA probe. DSB end resection beyond each restriction site for each time point in the ectopic recombination assay was estimated as a percentage of the signal intensity loss corresponding to the fragment of interest before break induction. In allelic recombination and in BIR assays both the HO cut chromosome and the homologous template chromosome have the same sequences and only the cut chromosome is being resected. In these two assays, therefore, we calculated DSB end resection beyond each restriction site as a percentage of the signal intensity corresponding to half of the signal of the fragment of interest before break induction. The donor choice assay was performed as previously described [30]. The probe used for detecting products of DSB repair was almost the entire *LYS2* ORF (*Xba*I – *Hin*dIII). The kinetics of product formation (repair) at each time point was determined by dividing the normalized intensity of the band corresponding to the product by the normalized intensity of the initial uncut band at time 0.

### Pulsed-field gel electrophoresis (PFGE)

To analyze DSB repair products in AM1003 derivative mutant strains, chromosomal DNA plugs were prepared and separated on a 1% agarose gel at 6 V/cm for 30 hrs (initial time = 10 s, final time = 35 s) by using the CHEF DRII apparatus (Bio-Rad), followed by Southern blotting and hybridization using a DNA fragment containing *ADE1* sequence as a probe.

### Detection of *de novo* telomere addition sites

To determine where *de novo* telomeres were added, PCR was carried out using one primer complementary to telomeric repeat sequence (CA16; 5′-CACCACACCCACACAC-3′) [57] and another primer at a different distance from the HO break site (Telo-F10 [∼5 kb upstream of HO]; 5′-GTCGTGCAGGTACGACTTTA-3′, Telo-F8 [∼4 kb upstream of HO]; 5′-TCTTTCCCTCGCTACTCACA-3′, Telo-F6 [∼3 kb upstream of HO]; 5′-GTGAGCGTACAAGAAGCAAA-3′, Telo-F2 [∼2 kb upstream of HO]; 5′-GTTAAGTAGTAAGTTTGCGGAG-3′, Telo-F4 [∼1 kb upstream of HO]; 5′-CCAACTTTCTAGTATTCGGACA-3′). Amplified PCR products were isolated from agarose gels and sequenced.

### Chromatin immunoprecipitation (ChIP)

ChIP analysis of Cdc13 binding was performed and quantification was done as described previously [5]. α-Myc antibody was purchased from Sigma (9E10). After immunoprecipitation and reverse crosslinking, SyBrGreen-based real-time PCR was carried out on an ABI 7900 machine using a pair of primers which anneal 1 kb upstream of the HO break site (MATX-F2, 5′-GGTAGGCGAGGACATTATCTATCA-3′; MATX-R3, 5′-GAAGAATACCAGTTTATCTCGCATTCAAATC-3′) as well as primers specific for the *PRE1* gene located on chromosome V as a control.

### Gene targeting efficiency and accuracy assay

An approximately 2.1 kb *thr4::* *URA3* cassette was PCR amplified from genomic DNA of the AM476 strain and 1 µg was used as a gene replacement construct for transformation of 1×10^8^ JKM139 cells and its derivative mutant cells. To calculate gene targeting efficiency, the number of Ura^+^ colonies was normalized by plating efficiency and transformation efficiency with uncut centromeric YCp50 plasmid for each strain. The accuracy of gene replacement was calculated as the number of Ura^+^ Thr^−^ transformants.

## Supporting Information

Figure S1 Kinetics of resection and HO induction in allelic recombination assay. (A) Schematic representation of allelic recombination assay between *MAT* **a** and *MAT* **a**-inc loci on chromosome III. Positions of *Eco*RI and *Hin*dIII sites and DNA probes used for Southern hybridization to analyze 5′ strand processing with respect to the HO recognition site are shown (yGI234). (B) Southern blotting analysis of resection and HO induction in allelic assay. The quantification is shown in Figure 2B. The same blot was probed subsequently with 8 different probes. Probes used for resection analysis recognize both the cut chromosome and the uncut homologous chromosome. Therefore, complete resection beyond each studied restriction fragment will eliminate only half of the studied restriction fragments. (C) Kinetics of HO break induction and DSB repair in allelic recombination assay.
(0.61 MB TIF)

Figure S2 DSB end resection in wild-type diploid cells. (A) The position of the restriction enzyme sites and the probes used to follow the resection kinetics in wild-type diploid cells at two different loci. (B) Southern blot analysis and plot demonstrating kinetics of resection are shown.
(0.24 MB TIF)

Figure S3 Kinetics of 5′ strand resection and repair in BIR assay. (A) Schematic representing BIR assay and the position of the restriction enzyme sites and the probe used to follow DSB repair via BIR. The probe used in this assay detects repair by BIR but not by gene conversion. (B) Southern blot analysis of HO break induction and repair in BIR assay. Quantification of repair and HO cut induction is shown. The kinetics of product formation in BIR assay at each time point was determined by subtracting the pixel intensity of the band corresponding to the initial (time 0) *MAT*α template DNA *Eco*RI fragment from the sum of the intensities of the bands corresponding to the template *MAT*α and the product DNA fragments multiplied by 100%. Quantities of DNA loaded on gels for each time point were normalized using a *TRA1* gene DNA probe. (C) Southern blot analysis of 5′ strand resection in BIR assay. Quantification of 5′ strand resection is shown in Figure 2.
(0.73 MB TIF)

Figure S4 Analysis of repair products in allelic recombination system. (A) Schematic representation of allelic recombination assay between *MAT* **a** and *MAT*α-inc loci on chromosome III. (B) The fate of the repair products was determined based on their auxotrophic markers in wild-type (yGI234) and *exo1*Δ *sgs1*Δ mutant (yWH847) cells. (C) To distinguish between BIR and de novo telomere formation, repair products from individual survivors that are Ade+ Thr- were analyzed by PFGE using an ADE1 probe. “0” indicates control before break induction. Position of chromosome I that carries the *ade1–1* gene is shown, as the parental strains used to make the diploid strain carry chromosome I of different sizes.
(0.47 MB TIF)

Figure S5 Pif1 helicase does not affect 5′ end resection at the DSBs. Comparison of the resection kinetics in wild-type, *exo1*Δ *sgs1*Δ and *pif1-*m2 *exo1*Δ *sgs1*Δ mutant cells at three different loci (0, 3, and 28 kb away from the HO break site using MAT, BUD5, and FEN2 probes, respectively). Southern blot analysis is shown.
(0.64 MB TIF)

Table S1 Genotype of strains used in the study.
(0.08 MB PDF)

Table S2 Sequences of DNA primers used in the study.
(0.04 MB PDF)
