# Supplementary figures and images for: Defective Resection at DNA Double-Strand Breaks Leads to De Novo Telomere Formation and Enhances Gene Targeting
Source: PLoS Genet. 2010 May 13;6(5):e1000948. doi: 10.1371/journal.pgen.1000948 (PMC2869328; doi:10.1371/journal.pgen.1000948)

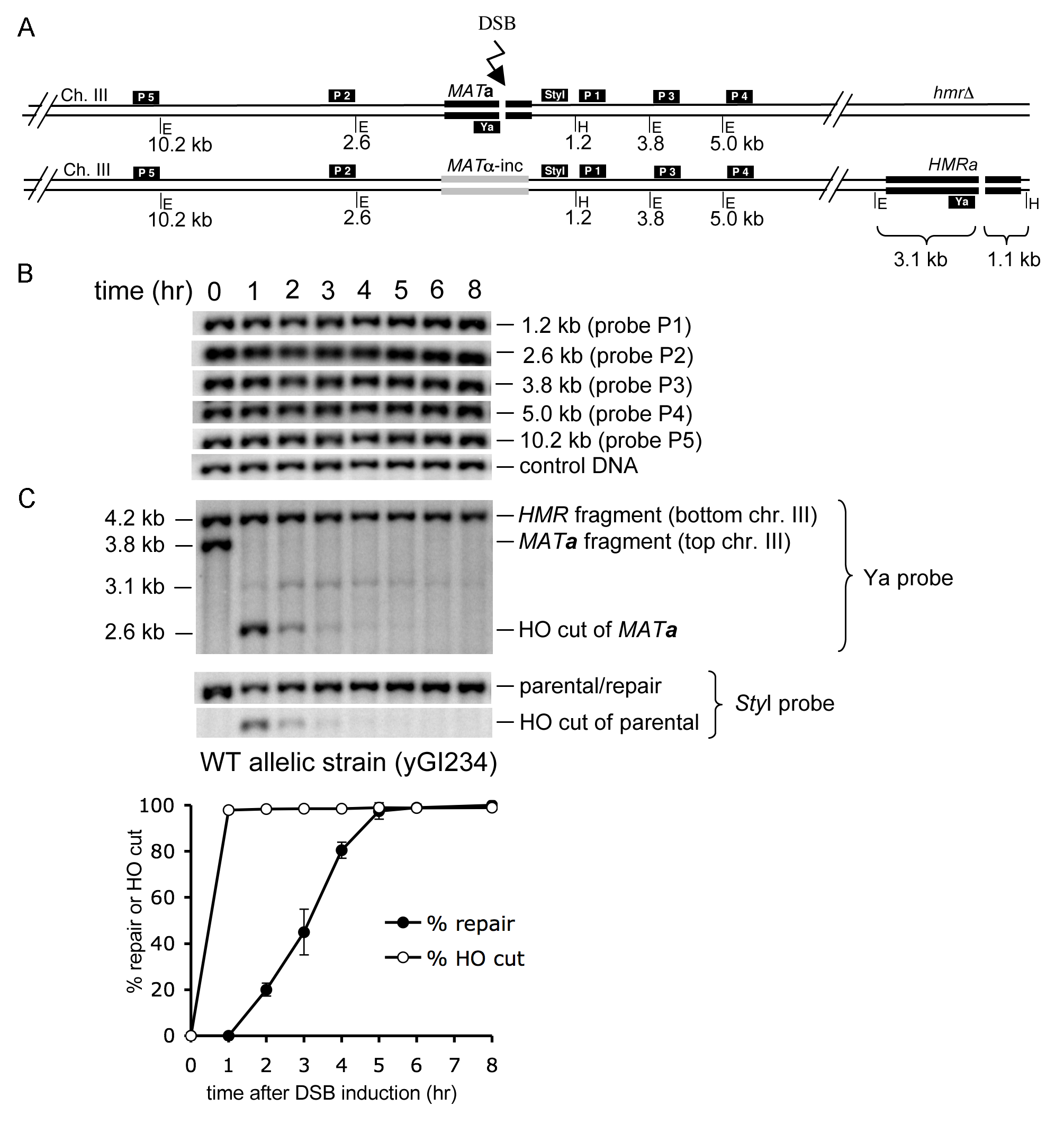

Supplement: Figure S1 — Kinetics of resection and HO induction in allelic recombination assay. (A) Schematic representation of allelic recombination assay between MAT a and MAT a-inc loci on chromosome III. Positions of EcoRI and HindIII sites and DNA probes used for Southern hybridization to analyze 5′ strand processing with respect to the HO recognition site are shown (yGI234). (B) Southern blotting analysis of resection and HO induction in allelic assay. The quantification is shown in Figure 2B. The same blot was probed subsequently with 8 different probes. Probes used for resection analysis recognize both the cut chromosome and the uncut homologous chromosome. Therefore, complete resection beyond each studied restriction fragment will eliminate only half of the studied restriction fragments. (C) Kinetics of HO break induction and DSB repair in allelic recombination assay. (0.61 MB TIF) [file pgen.1000948.s001.tif]

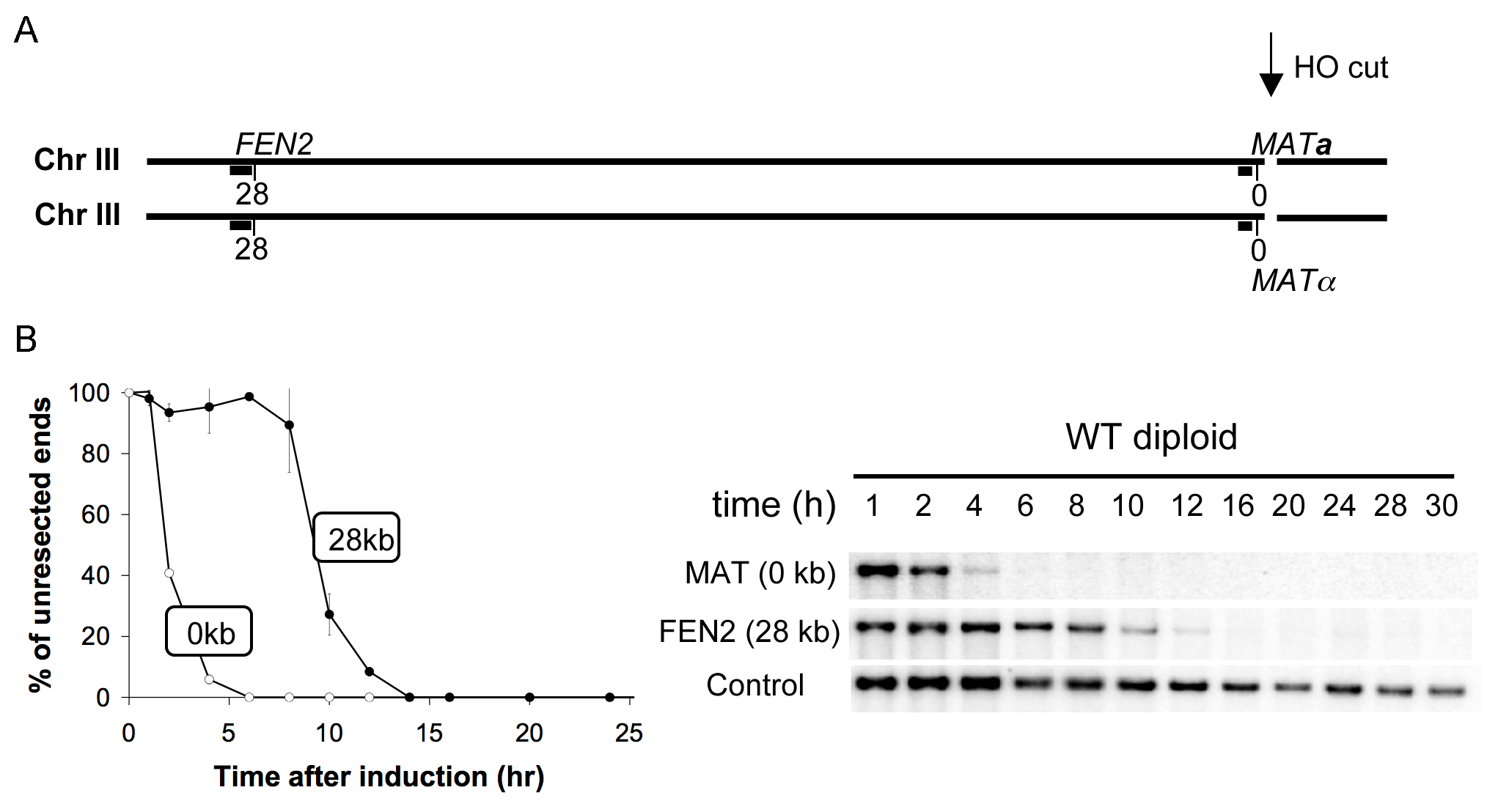

Supplement: Figure S2 — DSB end resection in wild-type diploid cells. (A) The position of the restriction enzyme sites and the probes used to follow the resection kinetics in wild-type diploid cells at two different loci. (B) Southern blot analysis and plot demonstrating kinetics of resection are shown. (0.24 MB TIF) [file pgen.1000948.s002.tif]

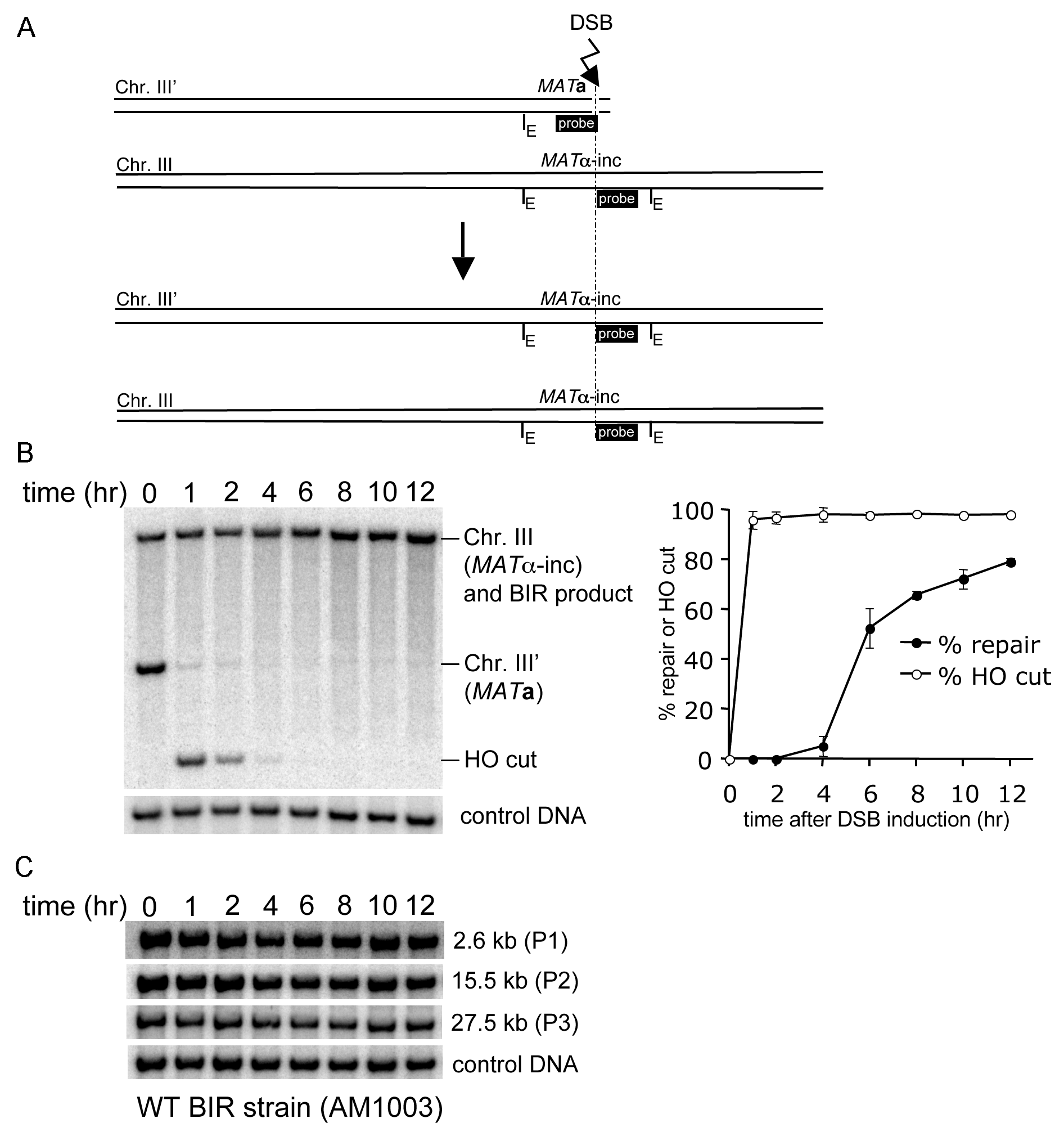

Supplement: Figure S3 — Kinetics of 5′ strand resection and repair in BIR assay. (A) Schematic representing BIR assay and the position of the restriction enzyme sites and the probe used to follow DSB repair via BIR. The probe used in this assay detects repair by BIR but not by gene conversion. (B) Southern blot analysis of HO break induction and repair in BIR assay. Quantification of repair and HO cut induction is shown. The kinetics of product formation in BIR assay at each time point was determined by subtracting the pixel intensity of the band corresponding to the initial (time 0) MATα template DNA EcoRI fragment from the sum of the intensities of the bands corresponding to the template MATα and the product DNA fragments multiplied by 100%. Quantities of DNA loaded on gels for each time point were normalized using a TRA1 gene DNA probe. (C) Southern blot analysis of 5′ strand resection in BIR assay. Quantification of 5′ strand resection is shown in Figure 2. (0.73 MB TIF) [file pgen.1000948.s003.tif]

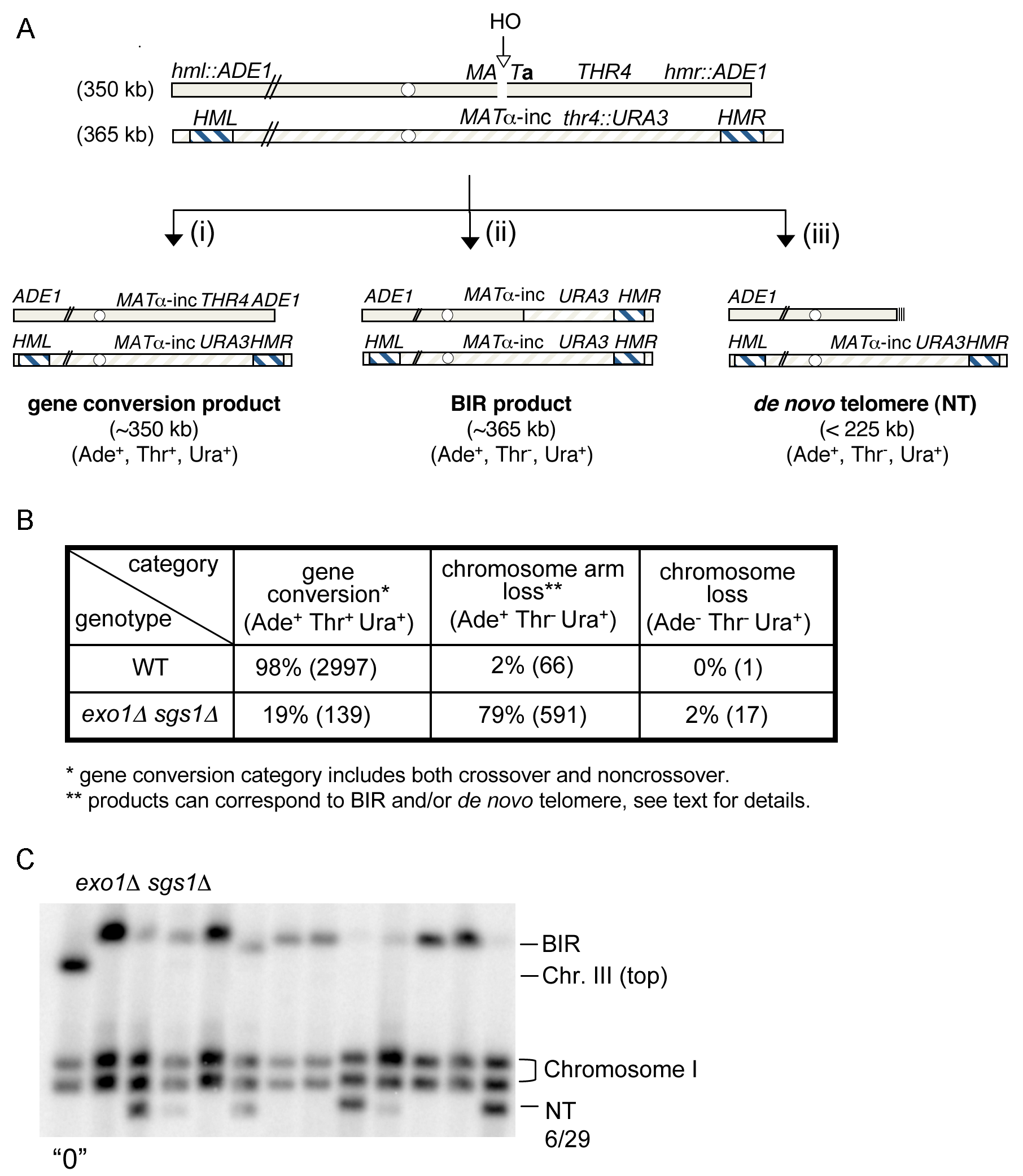

Supplement: Figure S4 — Analysis of repair products in allelic recombination system. (A) Schematic representation of allelic recombination assay between MAT a and MATα-inc loci on chromosome III. (B) The fate of the repair products was determined based on their auxotrophic markers in wild-type (yGI234) and exo1Δ sgs1Δ mutant (yWH847) cells. (C) To distinguish between BIR and de novo telomere formation, repair products from individual survivors that are Ade+ Thr- were analyzed by PFGE using an ADE1 probe. “0” indicates control before break induction. Position of chromosome I that carries the ade1–1 gene is shown, as the parental strains used to make the diploid strain carry chromosome I of different sizes. (0.47 MB TIF) [file pgen.1000948.s004.tif]

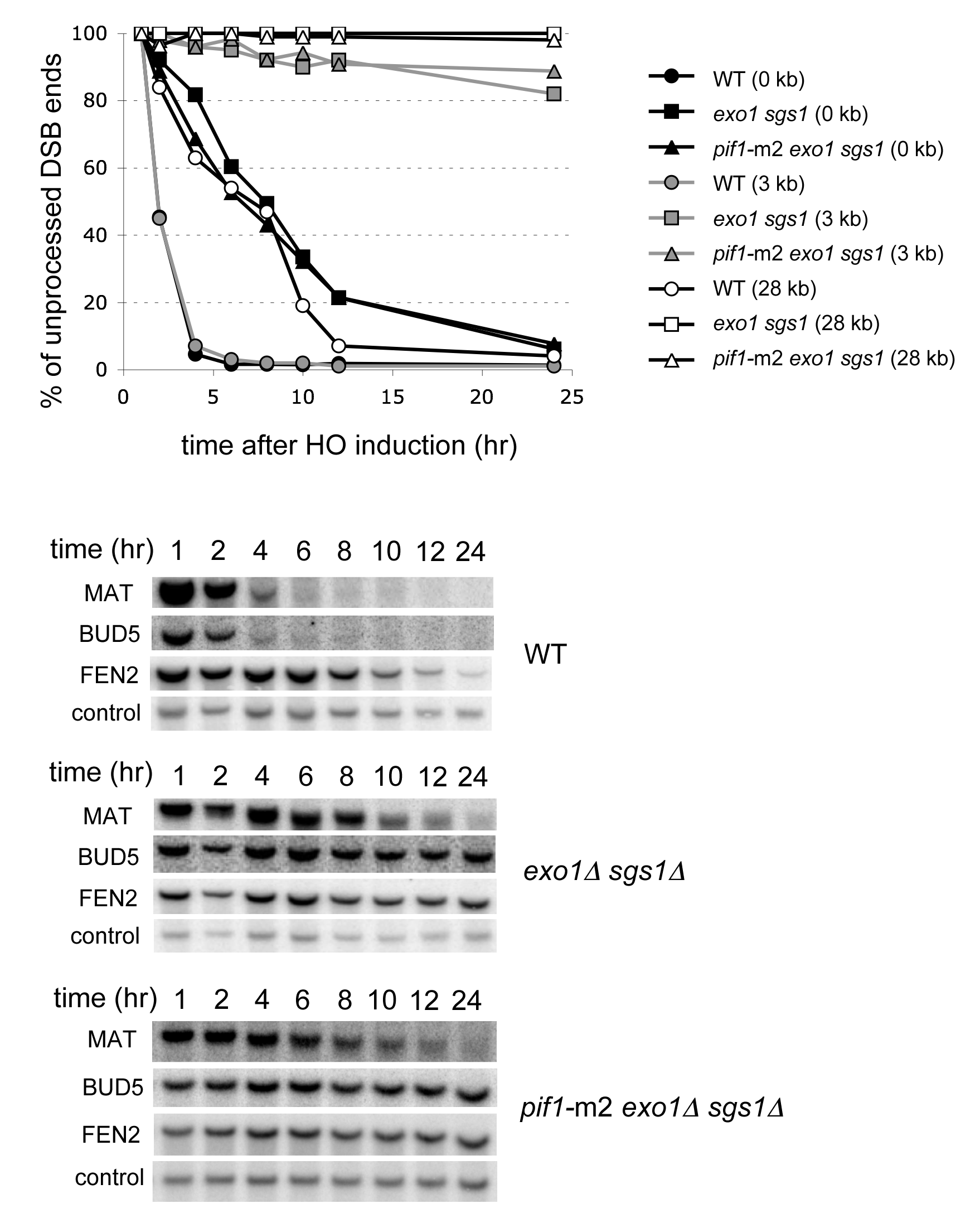

Supplement: Figure S5 — Pif1 helicase does not affect 5′ end resection at the DSBs. Comparison of the resection kinetics in wild-type, exo1Δ sgs1Δ and pif1-m2 exo1Δ sgs1Δ mutant cells at three different loci (0, 3, and 28 kb away from the HO break site using MAT, BUD5, and FEN2 probes, respectively). Southern blot analysis is shown. (0.64 MB TIF) [file pgen.1000948.s005.tif]
