# Supplementary material for: Defective Resection at DNA Double-Strand Breaks Leads to De Novo Telomere Formation and Enhances Gene Targeting
Source: PLoS Genet. 2010 May 13;6(5):e1000948. doi: 10.1371/journal.pgen.1000948 (PMC2869328; doi:10.1371/journal.pgen.1000948)
Supplement: Table S1 — Genotype of strains used in the study. (0.08 MB PDF) [file pgen.1000948.s006.pdf]

Table S1. Genotype of strains used in the study

strain name	parental strain	genotype	reference
tGI354	-	*hml::ADE1 MAT**a**-inc hmr::ADE1 ade1 leu2-3,112 lys5 trp1::hisG ura3-52 ade3::GAL::HO arg5,6::HPH::MAT**a***	[1]
tGI379	tGI354	*rad51::KanMX*	this study
yGI234	-	*hml::ADE1/HML MAT**a**/MATα-inc hmr::ADE1/HMR ade1/ade1 leu2-3,112/leu2-3,112 lys5/LYS5 MET13/met13 trp1::hisG/trp1 ura3-52/ura3 THR4/thr4::URA3 ade3::GAL10::HO/ADE3*	this study
yWH847	yGI234	*exo1::TRP1/exo1::TRP1 sgs1::KANMX/sgs1::KANMX*	this study
MK181	-	*MAT**a**-inc ura3-HOcs-inc ade3::GAL::HO ade2-1 leu2-3,112 his3-11,15 trp1-1 can1-100 LYS2::ura3-HOcs::LYS2 (5.6 kb) on chrII; LYS2 on chrXV*	[2]
yZZ233	MK181	*exo1::TRP1*	this study
yZZ240	MK181	*sgs1::KANMX*	this study
yZZ242	MK181	*exo1::TRP1 sgs1::KANMX*	this study
JKM139	-	*MAT**a** ho hml::ADE1 hmr::ADE1 ade1-100 leu2-3,112 lys5 trp1::hisG ura3-52 ade3::GAL10::HO*	[3]
yZZ115	JKM139	*exo1::TRP1 sgs1::KANMX*	this study
yZZ116	JKM139	*sgs1::KANMX*	this study
yZZ117	JKM139	*exo1::TRP1*	this study
yWH54	JKM139	*pif1-m2 dna2::KANMX*	this study
AM1003	-	*MAT**a**-LEU2-tel/MATα-inc ade1 met13 ura3 leu2-3,112/leu2 thr4 lys5 hml::ADE1/hml::ADE3 hmr::HPH ade3::GAL-HO FS2::NAT/FS2*	[4]
yWH121	AM1003	*pif1-m2*	this study
yWH576	AM1003	*exo1::TRP1*	this study
yWH578	AM1003	*sgs1::KANMX*	this study
yWH612	AM1003	*exo1::TRP1 sgs1::KANMX*	this study
yWH747	AM1003	*pif1-m2 exo1::TRP1 sgs1::KANMX*	this study
yWH942	AM1003	*pif1-m2 sml1::TRP1 mec1::KANMX*	this study
yWH786	AM1003	*yku70::KANMX exo1::TRP1 sgs1::URA3*	this study
yWH787	AM1003	*yku70::KANMX pif1-m2 exo1::TRP1 sgs1::URA3*	this study
yWH917	AM1003	*pif1-m1-4A::TRP1 pif1-m2 exo1::KANMX sgs1::URA3*	this study
yWH801	AM1003	*CDC13-13MYC-KANMX*	this study
yWH803	AM1003	*pif1-m2 CDC13-13MYC-KANMX*	this study

yWH805	AM1003	*exo1::TRP1 sgs1::URA3 CDC13-13MYC-KANMX*	this study
yWH807	AM1003	*pif1-m2 exo1::TRP1 sgs1::URA3 CDC13-13MYC-KANMX*	this study
AM476	-	*MATα-inc ade1 met13 ura3 leu2,3-112 trp1 thr4::URA3*	this study

1. Ira G, Malkova A, Liberi G, Foiani M, Haber JE (2003) Srs2 and Sgs1-Top3 suppress crossovers during double-strand break repair in yeast. Cell 115: 401-411.
2. Inbar O, Kupiec M (1999) Homology search and choice of homologous partner during mitotic recombination. Mol Cell Biol 19: 4134-4142.
3. Lee SE, Moore JK, Holmes A, Umezu K, Kolodner RD, et al. (1998) Saccharomyces Ku70, mre11/rad50 and RPA proteins regulate adaptation to G2/M arrest after DNA damage. Cell 94: 399-409.
4. Deem A, Barker K, Vanhulle K, Downing B, Vayl A, et al. (2008) Defective break-induced replication leads to half-crossovers in Saccharomyces cerevisiae. Genetics 179: 1845-1860.
