# Supplementary material for: Defective Resection at DNA Double-Strand Breaks Leads to De Novo Telomere Formation and Enhances Gene Targeting
Source: PLoS Genet. 2010 May 13;6(5):e1000948. doi: 10.1371/journal.pgen.1000948 (PMC2869328; doi:10.1371/journal.pgen.1000948)
Supplement: Table S2 — Sequences of DNA primers used in the study. (0.04 MB PDF) [file pgen.1000948.s007.pdf]

Table S2. Sequences of DNA primers used in the study.

figure	probe name	sequences	reference
Fig. 1A	P1	5’-GATCTGCAGCTCAGTCACATG-3’ (ARG5,6 distal 1F)<br>5’-ACTACCTACGCCAACCTATTC-3’ (ARG5,6 distal 1R)	this study
	P2	5’-GCATTAGGTTAAAGAGGCTC-3’ (ARG5,6 distal 2F)<br>5’-TGTTTGCTCAACCAAGTCAG-3’ (ARG5,6 distal 2R)	this study
	P3	5’-CATTACAGTGGTGCCACTTG-3’ (ARG5,6 distal 3F)<br>5’-CTAGCAATAACTATTCCCTC-3’ (ARG5,6 distal 3R)	this study
	P4	5’-CGTGATTCCAATGGATTTAG-3’ (ARG5,6 distal 4F)<br>5’-GGAAATGGCTTGCTTTGTGG-3’ (ARG5,6 distal 4R)	this study
Fig. 2A and Fig. S1	P1	5’-CAATTCCGTAAAGTCATAAG-3’ (Allelic HindIII dist F1)<br>5’-CTAGAAGACTCTAATATTCC-3’ (Allelic HindIII dist R1)	this study
	P2	5’-CCAGTTATCGTCCTACGTTC-3’ (BUD5 F1)<br>5’-GGTAAGCCTTGGAACCTTAG-3’ (BUD5 R1)	(Zhu et al., 2008)
	P3	5’-TCAAGCAAATCGACACATGG-3’ (Allelic Eco2 F1)<br>5’-GTATTATACAGCGACAATTCC-3’ (Allelic Eco2 R1)	this study
	P4	5’-CAGTAATAAGTCGTCCTGAG-3’ (Allelic EcoRI dist F1)<br>5’-CACTTGATGCTTCCTTATTG-3’ (Allelic EcoRI dist R1)	this study
	P5	5’-CTATCGATGGCTCTATAAGAC-3’ (SNT1 F1)<br>5’-CACGACTTATTGGACTAGTG-3’ (SNT1 R1)	(Zhu et al., 2008)
	Ya	5’-GTTCTTTCGGGGAAACTGTA-3’ (MAT Ya FP)<br>5’-CCACATTTCTTTGCAACTTC-3’ (MAT Ya RP)	this study
	StyI	5’-CTACAAAACTGTTGATTAGG-3’ (StyI FP)<br>5’-CTTTGATTAAGACTACTATC-3’ (StyI RP)	this study
Fig. 2C	P1	5’-CCAGTTATCGTCCTACGTTC-3’ (BUD5 F1)<br>5’-GGTAAGCCTTGGAACCTTAG-3’ (BUD5 R1)	(Zhu et al., 2008)
	P2	5’- CAAGATACTATAACAGGTGG-3’ (BPM1 F1)<br>5’- TGCCACTATACTATTTCCTC-3’ (BPM1 R1)	this study
	P3	5’-CACCAATGCATATATATCCG-3’ (FEN2 F1)<br>5’-GAATAGTCGACCAGTCTAAC-3’ (FEN2 R1)	(Zhu et al., 2008)
control	TRA1	5’-GTCCTAATACGACTTTTCAAATTGTCCTTTATGTCCGTCA-3’<br>5’-ATACTTGTAAGCACTCTTCCTGTAGTGAATATCACTTTTG-3’	(Zhu et al., 2008)
